# Unhealthy lifestyle factors and the risk of colorectal cancer: a Mendelian randomization study

**DOI:** 10.1038/s41598-024-64813-y

**Published:** 2024-06-15

**Authors:** Xingyuan Li, Zewen Chang, Jiaqi Wang, Ke Ding, Shengqi Pan, Hanqing Hu, Qingchao Tang

**Affiliations:** Department of Colorectal Surgery, The Second Affiliated Hospital of Harbin Medical University, 246 Xuefu Road, Harbin, China

**Keywords:** Mendelian randomization, Colorectal cancer, Obesity, Smoking, Lifestyle, Cancer prevention, Cancer therapy, Gastrointestinal cancer, Surgical oncology, Risk factors

## Abstract

The purpose of this study was to investigate the causal association between unhealthy lifestyle style factors and the risk of colorectal cancer, with the aim of preventing the occurrence of colorectal cancer by modifying unhealthy lifestyles. A two-sample Mendelian randomization (MR) approach was employed in this study, utilizing the inverse-variance weighted method as the primary research method. This MR analysis analyzed data of 3022 colorectal cancer cases and 174,006 controls from the FinnGen database. Single nucleotide polymorphisms (SNPs) associated with unhealthy lifestyle factors were selected as instrumental variables (IVs), including two obesity-related indicators, BMI (body mass index) and WHR (waist-to-hip ratio). Four phenotypes of smoking (smoking initiation, ever smoked, smoking per day, smoking cessation) and one phenotype of alcohol consumption (drinks per week). Four phenotypes of physical activity (accelerometer-based physical activity, moderate-to-vigorous physical activity, vigorous physical activity, strenuous sports or other exercises). All SNPs were obtained from published genome-wide association studies. The study found that the obesity-related indicator, higher WHR (OR = 1.38, 95% CI 1.12–1.70; P = 0.002) were associated with an increased risk of colorectal cancer, and two smoking phenotypes, cigarettes per day(OR = 1.30, 95% CI 1.01–1.68; P = 0.042)and smoking initiation (OR = 3.48, 95% CI 1.15–10.55; P = 0.028), were potentially associated with an increased risk of colorectal cancer. However, there was no evidence to suggest that physical activities and alcohol consumption were associated with colorectal cancer (all p > 0.05). In addition, the study detected no pleiotropy (all p > 0.05). This MR analysis indicates a causal association between a higher waist-to-hip ratio and the risk of colorectal cancer and a suggestive association between smoking and the risk of colorectal cancer among Europeans. These findings contribute to the understanding of the etiology of colorectal cancer and have potential implications for its prevention.

## Introduction

Colorectal cancer is among the most common malignant tumors worldwide. In 2020, global cancer statistics reported that colorectal cancer ranked second in incidence (10.0%) and third in mortality (9.4%) among 19.3 million new cancer cases and 9.9 million cancer deaths, respectively. Compared with 2018, both the incidence and mortality of colorectal cancer have shown an upward trend^1,2^. Despite the availability of numerous treatment modalities for colorectal cancer, encompassing endoscopic treatment, surgical treatment, radiotherapy, chemotherapy, and targeted therapy, the disease often manifests with conspicuous symptoms solely in its advanced stages. This latency in symptom presentation usually leads to a missed window for patients to receive optimal therapeutic intervention^3,4^. Therefore, it is particularly essential to identify the risk factors of colorectal cancer and to implement primary prevention strategies.

Various studies have shown that unhealthy lifestyle factors, such as unhealthy diet, smoking, alcohol consumption, obesity, and lack of physical activities, are associated with inflammatory responses, oxidative stress, and increased DNA methylation—essential mechanisms of cancer development^5^. In recent years, the relationship between unhealthy lifestyle factors and cancer has become a research hotspot. This interest stems from the fact that, compared with other etiologies, unhealthy habits can be modified through lifestyle improvements, thus playing an essential role in disease treatment and prevention. However, the results of various observational studies about the association between unhealthy lifestyle factors and the risk of colorectal cancer are inconsistent. For instance, a prospective cohort study on smoking and alcohol consumption showed that both were significantly associated with an increased risk of colorectal cancer^6^. In contrast, another case–control study concluded that moderate alcohol consumption could reduce the risk of colorectal cancer, defining moderate alcohol consumption as 12–35 g per day, which was significantly associated with a reduced risk of colorectal cancer (OR = 0.35, 95% CI 0.16–0.74)^7^. Similarly, studies on obesity and physical activities have also yielded controversial conclusions. This inconsistency arises because observational studies are susceptible to confounding bias and reverse causality, rendering them unreliable for causal inference^8,9^.

MR, which uses genetic variation as IVs to infer causal relationships, is second only to randomized controlled trials (RCTs) in the hierarchy of causal inference in evidence-based medicine. Since alleles follow the principle of random segregation and free combination during gamete formation, mendelian randomization can effectively avoid the influence of confounding bias and reverse causality when the three fundamental assumptions—strong association, independence, and exclusion restriction—are simultaneously satisfied. For this reason, mendelian randomization is also called “the natural randomized controlled trial”^10^. This study utilizes data obtained from published genome-wide association studies to employ Mendelian randomization in investigating the causal relationship between unhealthy lifestyle factors (obesity, smoking, alcohol consumption, physical activities) and the risk of colorectal cancer.

## Materials and methods

### Genetic variants associated with unhealthy lifestyle factors

In this study, we chose obesity, smoking, alcohol consumption, and physical activity as exposures. SNPs associated with obesity were obtained from a meta-analysis of GWAS for body fat distribution published in 2019, which included nearly 700,000 European individuals from the UK-Biobank and the Genetic Investigation of Anthropometric Traits (GIANT). Obesity indicators included BMI with 670 associated SNPs and WHR with 316 associated SNPs^11^. SNPs associated with smoking and alcohol consumption were sourced from a published meta-analysis of GWAS datasets summarized by Mengzhen Liu in 2019^12^, which included data from the UK biobank, 23andMe, and multiple epidemiological studies. This analysis included four phenotypes of smoking: smoking initiation (10 associated SNPs), ever smoked regularly (378 associated SNPs), cigarettes per day (55 associated SNPs), smoking cessation (24 associated SNPs), and one phenotype of alcohol consumption: drinks per week (99 associated SNPs). SNPs associated with physical activity were derived from a published meta-analysis of GWAS for habitual physical activity published in 2018^13^, including four phenotypes of physical activities: accelerometer-based physical activity (8 associated SNPs), moderate-to-vigorous physical activity (19 associated SNPs), vigorous physical activity (7 associated SNPs), and strenuous sports or other exercises (14 associated SNPs). Detailed information about these datasets can be found in Table 1. In addition, in our study, to ensure the robustness of our results, SNPs associated with the exposure must meet the GWAS threshold of p < 5 × 10^–8^, and SNPs must demonstrate a strong correlation with the exposure (F > 10)^14^. Additionally, linkage disequilibrium analysis was conducted to ensure complete independence between SNPs (r^2^ < 0.001, kb > 10,000). Finally, palindrome sequences were excluded as they can affect gene expression.

**Table 1:** Details on the characteristics of each included dataset.

Phenotype	Data source	Total sample size	Population	Consortium
Obesity-related-traits				
BMI	Pulit SL, Stoneman C, Morris AP et al. Meta-analysis of genome-wide association studies for body fat distribution in 694 649 individuals of European ancestry. Hum Mol Genet. 2019 Jan 1;28(1):166-174.	694, 648	European	UK-Biobank and GIANT
WHR				
Smoking and drinking-related-traits				
AgeSmk	Liu M, Jiang Y, Wedow R. et al. Association studies of up to 1.2 million individuals yield new insights into the genetic etiology of tobacco and alcohol use. Nat Genet. 2019 Feb;51(2):237-244.	1,232,091	European	UK-Biobank and 23 and me
CigDay				
SmkInit				
SmkCes				
DrnkWk				
Physical activity-related-traits				
AccAve	Klimentidis YC, Raichlen DA, Bea J, Garcia DO, Wineinger NE, Mandarino LJ, et al. Genome-wide association study of habitual physical activity in over 377,000 UK Biobank participants identifies multiple variants including Cadm2 and Apoe. Int J Obes. 2018;42(6):1161–1176. 10.1038/s41366-018-0120-3	377,234	European	UK-Biobank and ARIC
MVPA				
VPA				
SSOE				
Colorectal cancer	https://r9.finngen.fi/pheno/C3_COLORECTAL_EXALL	3022 cases 174,006 controls	European	FinnGen

*AgeSmk* age of initiation of regular smoking, *SmkInit* a binary phenotype indicating whether an individual had ever smoked regularly, *CigDay* heaviness of smoking was measured with cigarettes per day, *SmkCes* smoking cessation, *DrnkWk* available measures of alcohol use were simpler, with drinks per week, *AccAve* accelerometer-based physical activity measurement (average acceleration), *MVPA* moderate-to-vigorous physical activity, *SSOE* strenuous sports or other exercise, *VPA* vigorous physical activity, *BMI* body mass index, *WHR* waist-to-hip ratio, *WHRadjBMI* waist-to-hip ratio adjusted for body mass index.

### GWAS summary data for colorectal cancer

The GWAS summary data for colorectal cancer were obtained from the FinnGen database (https://r9.finngen.fi/pheno/C3_COLORECTAL_EXALLC), including 3022 colorectal cancer cases and 174,006 controls of European ancestry, ensuring no sample overlap between exposures and outcome. Detailed data sources are presented in Table 1.

### Statistical analyses

**Figure 1:**
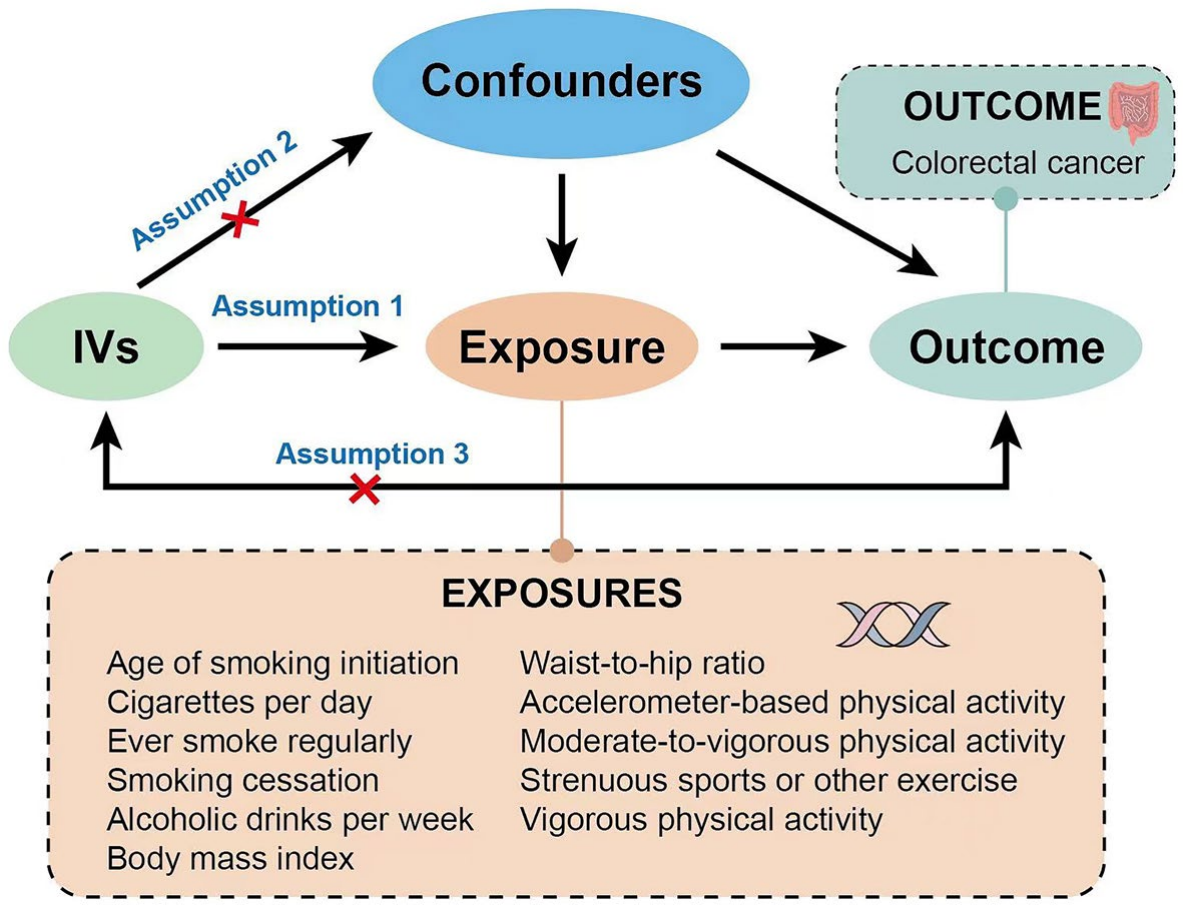
Overview and assumptions of the Mendelian randomization study. Assumption 1 (Relevance assumption): IVs need to have a stable and robust correlation with the exposure; Assumption 2 (Independence assumption): IVs are independent of any confounders affecting the exposure-outcome relationship; Assumption 3 (Exclusiveness assumption): IVs can only affect the outcome indirectly through the exposure, and not through other approaches.

In this study, we utilized the two-sample Mendelian randomization to investigate the causal association between unhealthy lifestyle factors and the risk of colorectal cancer. Mendelian randomization is a particular study method that uses genetic variation as IVs. Three main assumptions must be met: Assumption 1 (Relevance assumption): IVs must have a stable and robust correlation with the exposure; Assumption 2 (Independence assumption): IVs must be independent of any confounders affecting the exposure-outcome relationship; Assumption 3 (Exclusiveness assumption): IVs can only affect the outcome indirectly through the exposure, not through other pathways (Fig. 1)^15^.

We utilized inverse-variance weighting (fixed effects and multiplicative random effects), MR-Egger, Weighted median method, MR-pleiotropy residual sum and outlier (MR-PRSESSO to estimate the causal relationship between exposure and outcome. The IVW method was used as the primary research method. When heterogeneity was absent, we used the fixed effects model as the primary method; when heterogeneity was present, we used the random effects model as the primary method^16^. As supplementary, MR-Egger, Weighted median method, and MR-PRSESSO were employed to test the consistency of causality^16–18^. In sensitivity analysis, we used Cochran’s Q test to estimate heterogeneity and MR-Egger regression analysis to detect horizontal pleiotropy. We also utilized MR-PRESSO to identify and remove abnormal SNPs and calculate whether the results changed after removing outliers. Finally, the leave-one-out method was used to verify whether a single SNP drove the results. Lastly, to ensure the robustness of our results, we also performed multiple testing corrections using the Bonferroni method. Since this study investigates 11 exposure-outcome pairs, we set our p-value threshold at 0.05/11. The results of this study were deemed meaningful, satisfying criteria such as a P-value less than 0.05/11 in the IVW method, consistent directionality of beta values across various methods, and the absence of horizontal pleiotropy; when the p-value falls between 0.05/11 and 0.05, we consider the association between the exposure and the outcome to be suggestive. Our MR analyses were performed using the TwoSampleMR package (version 0.5.7) and MR-PRESSO package (version 1.0) in R (version 4.2.3).

## Results

**Figure 2:**
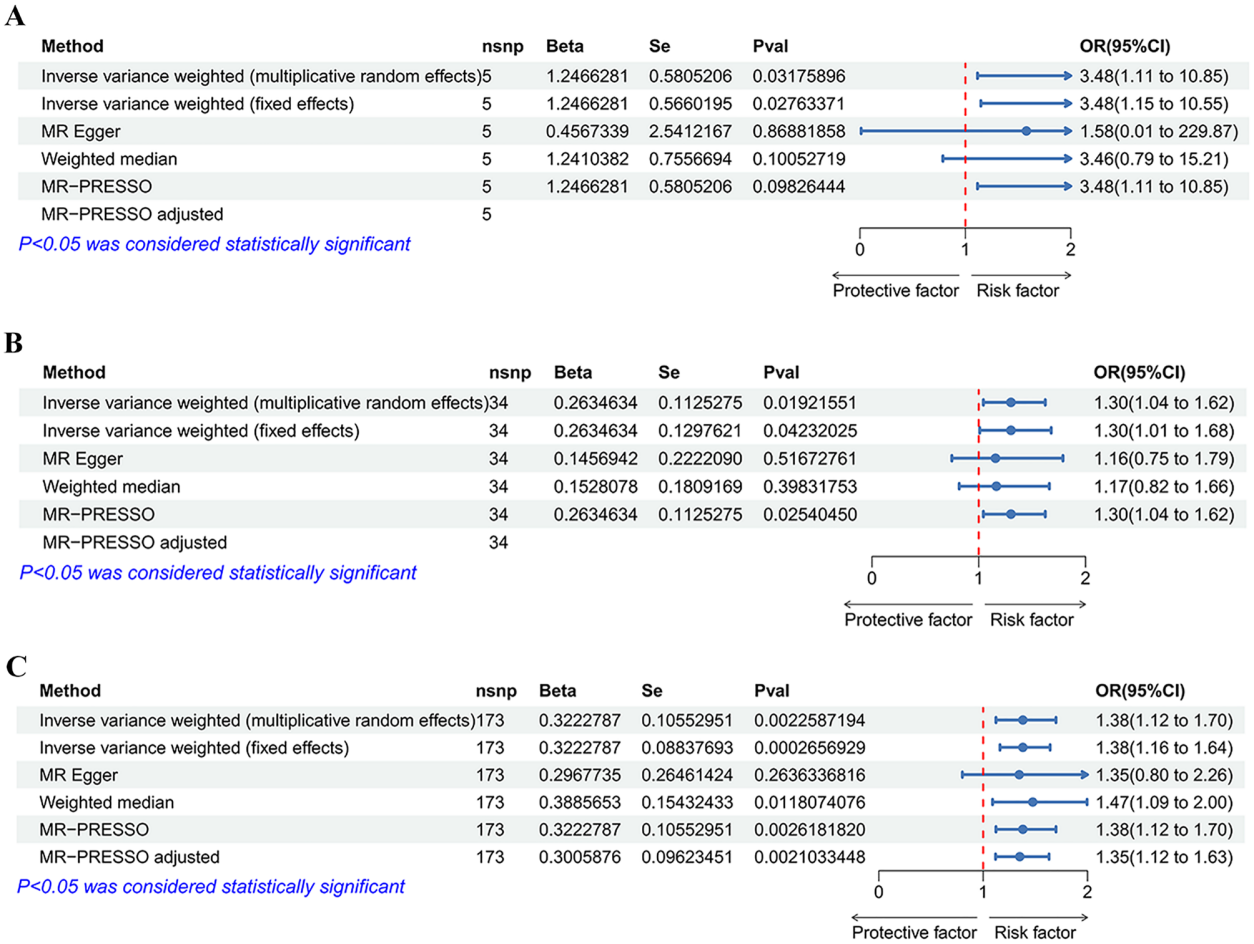
The forest plots depict a causal association between smoking initiation, cigarettes per day, Waist-to-hip ratio and colorectal cancer. IVW method was regarded as the primary method in this study. (**A**) Age of initiation of regular smoking; (**B**) cigarettes per day; (**C**) waist-to-hip ratio.

**Figure 3:**
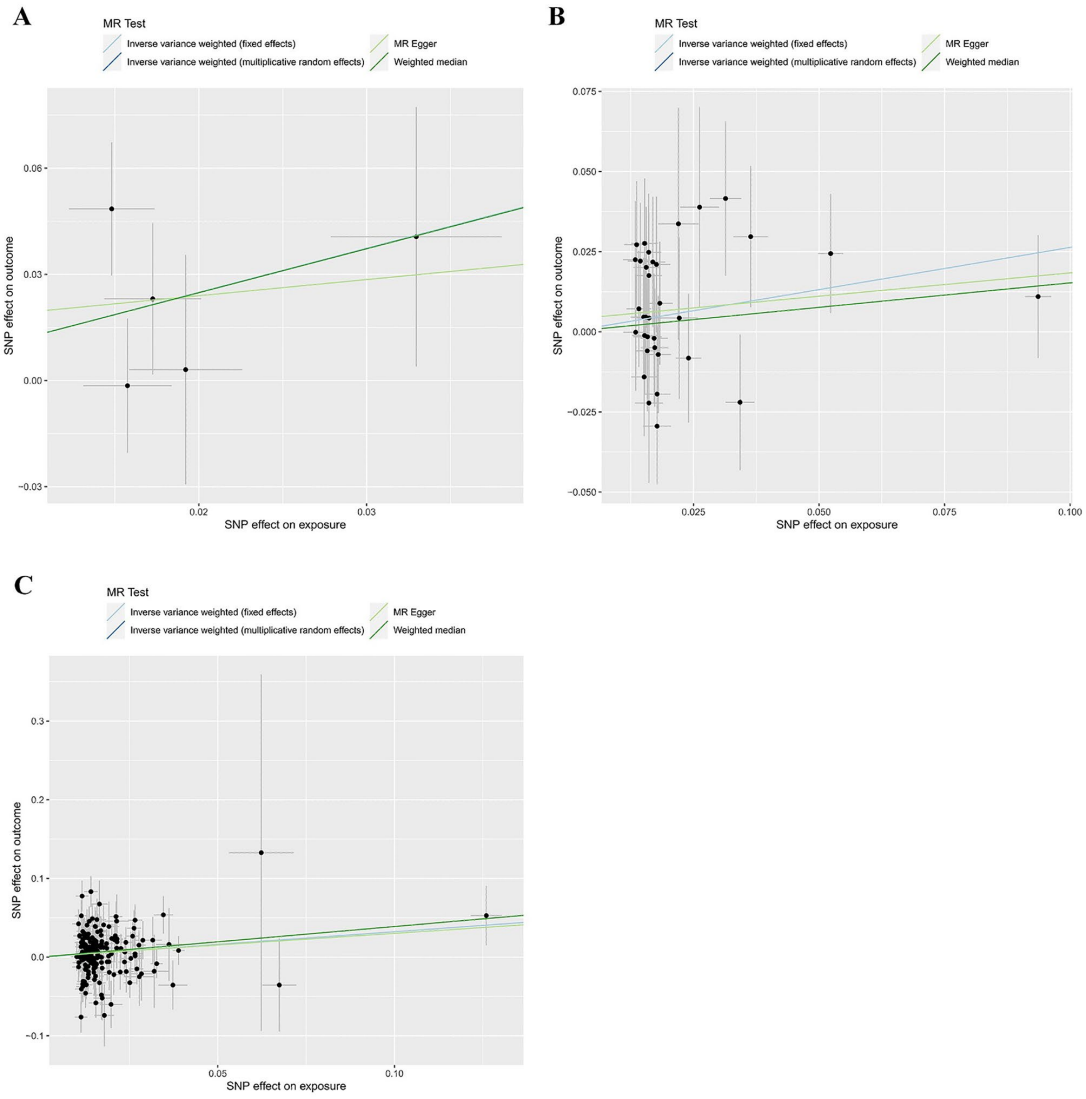
The Scatter plots depict a causal association between smoking initiation, cigarettes per day, Waist-to-hip ratio and colorectal cancer. (**A**) Age of initiation of regular smoking; (**B**) cigarettes per day; (**C**) waist-to-hip ratio.

**Figure 4:**
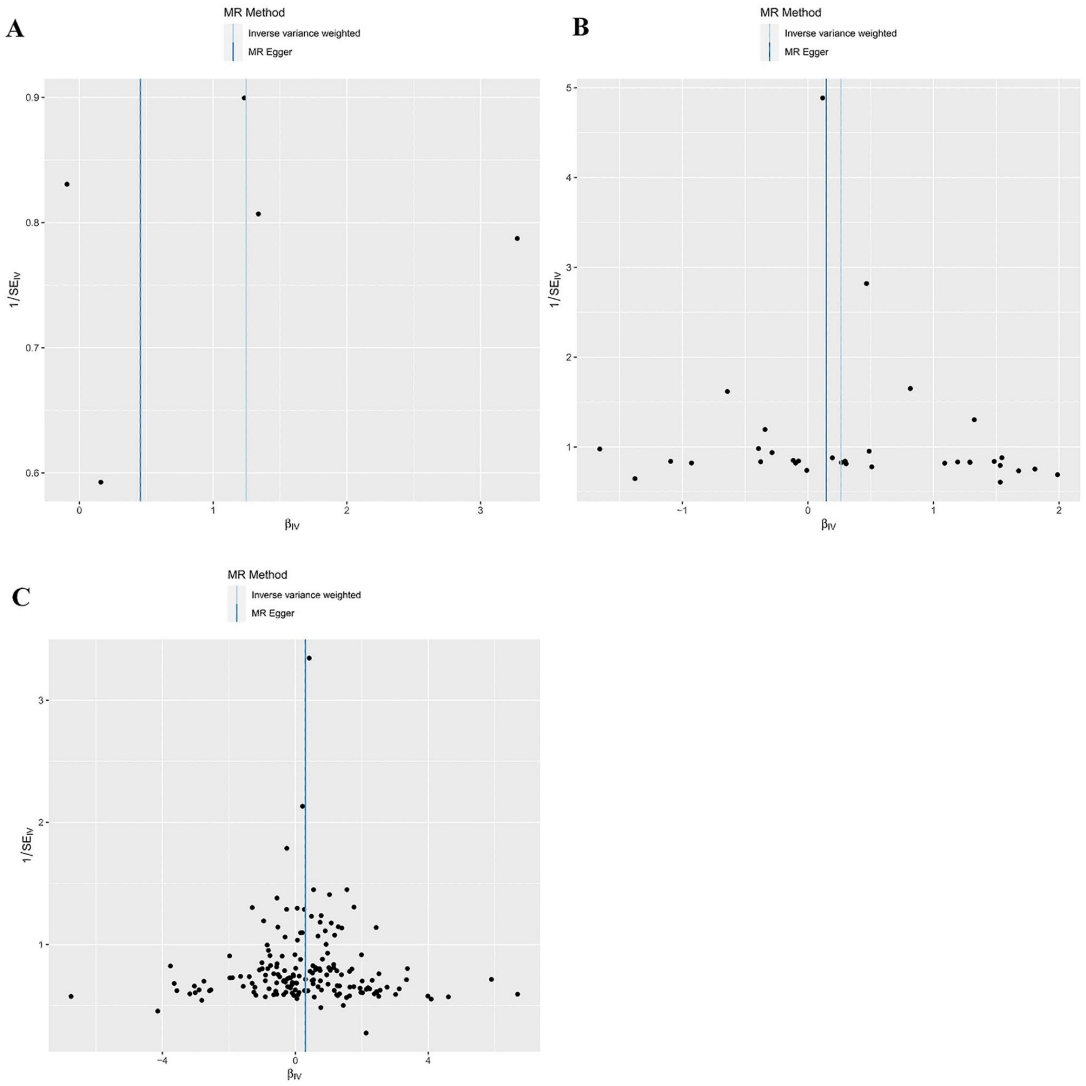
The funnel plots for evaluating heterogeneity. The blue line indicates inverse variance weighted assessment, while the dark blue line represents MR-Egger. (**A**) Age of initiation of regular smoking, (**B**) cigarettes per day, (**C**) waist-to-hip ratio

In the heterogeneity test, the P value for Cochran’s Q test of SNPs associated with smoking initiation and cigarettes per day were 0.379 and 0.846, respectively (Table 4). This result indicated no heterogeneity, so we chose IVW (fixed effects) as the primary method. Genetically predicted smoking initiation (OR = 3.48, 95% CI 1.15–10.55; P = 0.028) and cigarettes per day (OR = 1.30, 95% CI 1.01–1.68; P = 0.042) were suggestively associated with an increased risk of colorectal cancer (Table 2 and Figs. 2, 3). In MR-Egger regression analyses, the P values for intercept were 0.769 and 0.518, indicating no horizontal (Table 4); MR-PRESSO did not identify any outliers, and the leave-one-out method showed that the results of this MR analysis were not driven by any single SNP (Fig. S1). The funnel plot indicated a fundamentally symmetrical dispersion of the causal associations, underscoring the absence of discernible bias in the results (Fig. 4). Moreover, the direction of the beta values was consistent across all research methods, confirming the robustness of the results.

**Table 2:** Different methods of MR of the associations between unhealthy lifestyle factors and colorectal cancer.

Exposure	IVW (fixed effects)		IVW (multiplicative random effects)		MR-Egger	
	OR (95% CI)	*P*-value	OR (95% CI)	*P*-value	OR (95% CI)	*P*-value
Smoking						
AgeSmk	3.48 (1.15, 10.55)	0.028	3.48 (1.11, 10.85)	0.032	1.58 (0.01, 229.87)	0.869
CigDay	1.30 (1.01, 1.68)	0.042	1.30 (1.04, 1.62)	0.019	1.16 (0.75, 1.79)	0.518
SmkInit	0.86 (0.74, 1.00)	0.055	0.86 (0.73, 1.02)	0.078	0.70 (0.36, 1.37)	0.303
SmkCes	1.02 (0.70, 1.48)	0.929	1.02 (0.73, 1.41)	0.918	0.92 (0.31, 2.75)	0.888
Alcohol consumption						
DrnkWk	0.75 (0.49, 1.17)	0.209	0.75 (0.44, 1.30)	0.306	1.46 (0.40, 5.32)	0.572
Obesity						
BMI	1.12 (0.98, 1.29)	0.100	1.12 (0.97, 1.30)	0.124	1.19 (0.83, 1.69)	0.342
WHR	1.38 (1.16, 1.64)	0.000	1.38 (1.12, 1.70)	0.002	1.35 (0.80, 2.26)	0.264
Physical activity						
AccAve	0.97 (0.90, 1.05)	0.468	0.97 (0.87, 1.09)	0.616	1.20 (0.77, 1.87)	0.461
MVPA	0.59 (0.31, 1.11)	0.104	0.59 (0.29, 1.19)	0.140	0.12 (0.00, 6.47)	0.310
VPA	0.36 (0.08, 1.71)	0.199	0.36 (0.09, 1.37)	0.135	0.55 (0.00, 95,849.97)	0.927
SSOE	1.84 (0.41, 8.24)	0.427	1.84 (0.51, 6.57)	0.349	0.05 (0.00, 66.90)	0.439

*AgeSmk* age of initiation of regular smoking, *SmkInit* a binary phenotype indicating whether an individual had ever smoked regularly, *CigDay* heaviness of smoking was measured with cigarettes per day, *SmkCes* smoking cessation, *DrnkWk* available measures of alcohol use were simpler, with drinks per week, *AccAve* accelerometer-based physical activity measurement (average acceleration), *MVPA* moderate-to-vigorous physical activity, *SSOE* strenuous sports or other exercise, *VPA* vigorous physical activity.

For WHR, the heterogeneity test showed that heterogeneity existed (P value for Cochran’s Q test < 0.001) (Table 4), leading us to opt for IVW (multiplicative random effects) as the primary study method. Genetically predicted higher WHR was significantly associated with an increased risk of colorectal cancer (OR = 1.38, 95% CI 1.12–1.70; P = 0.002). Complementary analytical methods yielded consistent results: OR = 1.47, 95% CI 1.09–2.00; P = 0.012 by Weighted median method; OR = 1.38, 95% CI 1.12–1.70; P = 0.003 by MR-PRSESSO; OR = 1.35, 95% CI 0.80–2.26; P = 0.264 by MR-Egger. The MR-Egger result was insignificant, likely due to its insufficient statistical power and susceptibility to outliers (Tables 2, 3 and Figs. 2, 3). MR-Egger regression showed no evidence of horizontal pleiotropy (P value for intercept = 0.916) (Table 4). In MR-PRESSO, we identified and removed outliers and re-performed the MR analysis, and the results still suggested that higher WHR was associated with the increased risk of colorectal cancer ([OR], 95% CI 1.35, 1.12–1.63; P = 0.002). The leave-one-out method confirmed that the results of this MR analysis were not driven by any single SNP (Fig. S1). Additionally, the symmetrical funnel plot further indicated the robustness of the results (Fig. 4).

**Table 3:** Different methods of MR of the associations between unhealthy lifestyle factors and colorectal cancer.

Exposure	Weighted median method		MR-PRESSO		MR-PRESSO adjusted	
	OR (95% CI)	*P*-value	OR (95% CI)	*P*-value	OR (95% CI)	*P*-value
Smoking						
AgeSmk	3.46 (0.79,15.21)	0.101	3.48 (1.11,10.85)	0.098		
CigDay	1.17 (0.82,1.66)	0.398	1.30 (1.04,1.62)	0.025		
SmkInit	0.86 (0.69,1.09)	0.208	0.86 (0.73,1.02)	0.080		
SmkCes	0.85 (0.51,1.42)	0.530	1.02 (0.73,1.41)	0.921		
Alcohol consumption						
DrnkWk	1.01 (0.47,2.15)	0.981	0.75 (0.44,1.30)	0.310	0.83 (0.50,1.37)	0.464
Obesity						
BMI	1.12 (0.87,1.45)	0.373	1.12 (0.97,1.30)	0.125		
WHR	1.47 (1.09,2.00)	0.012	1.38 (1.12,1.70)	0.003	1.35 (1.12,1.63)	0.002
Physical activity						
AccAve	0.96 (0.86,1.07)	0.450	0.97 (0.87,1.09)	0.637		
MVPA	0.43 (0.18,1.04)	0.061	0.59 (0.29,1.19)	0.158		
VPA	0.19 (0.03,1.38)	0.101	0.36 (0.09,1.37)	0.185		
SSOE	1.06 (0.13,8.90)	0.955	1.84 (0.51,6.57)	0.369		

*AgeSmk* age of initiation of regular smoking, *SmkInit* a binary phenotype indicating whether an individual had ever smoked regularly, *CigDay* heaviness of smoking was measured with cigarettes per day, *SmkCes* smoking cessation, *DrnkWk* available measures of alcohol use were simpler, with drinks per week, *AccAve* accelerometer-based physical activity measurement (average acceleration), *MVPA* moderate-to-vigorous physical activity, *SSOE* strenuous sports or other exercise, *VPA* vigorous physical activity.

**Table 4:** Sensitivity test of MR of the associations between unhealthy lifestyle factors and colorectal cancer.

Exposure	MR-Egger regression analysis		Cochran’s Q test	
	Intercept	*P*-value	Q statistic	*P*-value
Smoking				
AgeSmk	0.015	0.769	4.21	0.379
CigDay	0.004	0.518	24.82	0.846
SmkInit	0.004	0.543	200.82	0.048
SmkCes	0.004	0.856	6.810	0.008
Alcohol consumption				
DrnkWk	− 0.009	0.278	87.34	0.310
BMI	− 0.001	0.733	308.89	0.052
WHR	0.000	0.916	245.24	0.000
Physical activity				
AccAve	− 0.060	0.386	10.45	0.064
MVPA	0.024	0.433	20.62	0.244
VPA	− 0.004	0.947	4.43	0.619
SSOE	0.029	0.343	7.92	0.720

*AgeSmk* age of initiation of regular smoking, *CigDay* heaviness of smoking was measured with cigarettes per day, *SmkInit* a binary phenotype indicating whether an individual had ever smoked regularly, *SmkCes* smoking cessation, *DrnkWk* available measures of alcohol use were simpler, with drinks per week, *AccAve* accelerometer-based physical activity measurement (average acceleration), *MVPA* moderate-to-vigorous physical activity, *SSOE* strenuous sports or other exercise, *VPA* vigorous physical activity.

## Discussion

To date, the prevention of colorectal cancer remains a challenging issue. In this study, we utilized a two-sample Mendelian randomization to investigate the causal association between unhealthy lifestyle factors and the risk of colorectal cancer in European populations. The results suggest that a higher WHR is associated with an increased risk of colorectal cancer, and smoking is potentially associated with the incidence of colorectal cancer. However, no definitive evidence was found to support an association between alcohol consumption and physical activity with the incidence of colorectal cancer.

### Smoking

Our study found a potential association between smoking and an increased risk of colorectal cancer. A systematic review of six cohort studies and fifteen case–control studies in the Japanese population suggested that smoking may contribute to the increased incidence of rectal cancer, but there was insufficient epidemiologic evidence to prove an association between smoking and colon cancer^19^. Another prospective study, which included 120,000 Cubans, investigated the relationship between different smoking ages and premature mortality. The results showed that smoking could lead to an increased incidence of various digestive tumors, including colorectal cancer, with the effect being most pronounced in individuals who began smoking before the age of 10. The risk of premature mortality in these individuals was approximately 2.51 times that of non-smokers^20^. A meta-analysis of 160 observational studies examining the association between smoking and the incidence and mortality of colorectal cancer concluded that smoking was significantly associated with the incidence of colorectal cancer, with a more pronounced effect in rectal cancer^21^. Despite these findings, the specific mechanism by which smoking mediates colorectal cancer remains unclear. A recent study indicated that smoking may increase the intestinal levels of taurodeoxycholic acid (TADC) by inducing gut microbiota dysbiosis. TADC can activate signaling pathways such as MAPK/ERK, IL-17, and TNF, leading to tumorigenesis. Furthermore, intestinal barrier dysfunction caused by gut microbiota dysbiosis could further facilitate this process^22^. Our Mendelian randomization analysis suggests a possible association between smoking and colorectal cancer, emphasizing the importance of tobacco control in reducing the long-term burden of this disease.

### Alcohol consumption

Our study did not find a causal association between alcohol consumption and the risk of colorectal cancer. This conclusion contrasts with the results of various observational studies. A case–control study examining the relationship between alcohol consumption and colorectal cancer risk in the Mediterranean population concluded that moderate alcohol consumption (12–25 g/day) was a protective factor for colorectal cancer (OR = 0.35; 95% CI 0.16–0.74), while heavy alcohol consumption (more than 48 g/day) was a risk factor for colorectal cancer (OR = 3.45; 95% CI 1.35–8.83). This effect was closely related to the type of wine consumed, with moderate red wine consumption thought to reduce the risk of many types of cancer, including colorectal cancer^7^. Additionally, a meta-analysis of five case–control studies and eleven prospective nested case–control studies involving 14,276 colorectal cancer cases and 15,802 controls supported this conclusion^23^. However, a cohort study of dietary inflammatory potential and the risk of colorectal cancer showed that for some specific populations (pro-inflammatory diet), abstaining from alcohol was a risk factor for colorectal cancer (OR = 1.02, P = 0.002)^24^. Although our MR analysis found no causal association between alcohol consumption and colorectal cancer, the potential effect of alcohol consumption on colorectal cancer cannot be entirely excluded. Our study focused solely on a European population, so further research is necessary to confirm these findings.

### Physical activity

While we did not find a causal association between physical activity and colorectal cancer in this study, the role of physical activity in cancer prevention and treatment is well established. A recently published review on the mechanism of exercise in cancer prevention and therapy suggested that physical activity could reduce cancer risk by inhibiting tumor cell proliferation, regulating tumor metabolism and the immune microenvironment, and inducing apoptosis^25^. Numerous observational studies have also concluded that physical activity reduces the risk of colorectal cancer^26–30^. Therefore, despite the uncertainty in our MR analysis regarding the causal association between physical activity and colorectal cancer, the potential benefits of exercise should not be overlooked. Future studies with larger sample sizes are warranted to provide a more comprehensive MR analysis.

### Obesity

Obesity has been defined by the International Agency for Research on Cancer (IARC) as a risk factor for colorectal cancer^31,32^, a view confirmed by numerous observational studies. BMI and WHR are both used to evaluate obesity. BMI primarily assesses overall obesity, while WHR evaluates central obesity. BMI is currently the most commonly used indicator to measure obesity. However, a cohort study including 387,672 UK participants concluded that WHR is more powerful and robust in predicting morbidity and mortality than BMI^33^. A review summarizing the epidemiological and pathophysiological evidence on the relationship between obesity and cancer showed that WHR was more strongly associated with the risk of cancer than BMI^34^. In addition, numerous studies have demonstrated that WHR has a stronger prediction ability than BMI in diseases such as diabetes, prostate cancer, cardiovascular risk, cirrhosis, and gastroesophageal reflux disease^35–40^. For colorectal cancer, central obesity is more closely associated with the disease than overall obesity. A cohort study of 134,255 Chinese participants investigating the association between weight, fat distribution, and colorectal cancer risk concluded that although both BMI and WHR were significantly associated with the risk of colorectal cancer, in the analyses stratified by WHR and BMI, the positive association between BMI and CRC weakened while that of WHR was still significant^41^. In this study, we further confirm the causal association between higher WHR and an increased risk of colorectal cancer through two-sample Mendelian randomization. This conclusion suggests that fat distribution should be the focus of future studies on the association between obesity and colorectal cancer rather than total fat.

Our study has several strengths: firstly, we utilized two-sample Mendelian randomization as the study method, which ranks second only to RCTs in the level of causal inference of evidence-based medicine. This method avoids the influence of confounding bias and reverse causality, making the inference of causality more reliable. Additionally, Mendelian randomization is less costly and more accessible than RCTs. Secondly, the SNPs used in our study are all obtained from published genome-wide association study data analysis, which ensures a strong correlation between SNPs and exposure. Thirdly, the data used in this study are all from populations of European ancestry, and the SNPs for exposure and outcome were derived from different sources, thereby avoiding population stratification bias and sample overlap.

Our study also has some limitations. Firstly, our research objectives are limited to the European population, necessitating further research to determine if the results are consistent across other racial groups. Additionally, the impact of unhealthy lifestyle factors on colorectal cancer may vary across different anatomical sites, pathological subtypes, and genders. However, due to the limitations in data availability, there are no suitable GWAS colorectal cancer datasets available for conducting subgroup analyses. A more comprehensive study including different subgroups of colorectal tumors will be implemented in the future. Secondly, the occurrence of most diseases, especially cancers, requires the synergistic effect of multiple genes and environmental factors. Mendelian randomization can only investigate the influence of a single gene and cannot comprehensively consider the complexity of multi-gene synergy. In addition, some potential confounders between the exposure and outcome and an insufficient sample size may bias the conclusion. Therefore, a large number of follow-up studies are needed to further refine the findings of this study.

All in all, this study indicated that higher WHR is a risk factor for colorectal cancer, and smoking is potentially associated with the colorectal cancer risk. From the perspective of basic and clinical research, we suggest that future research on colorectal cancer should not only consider BMI but also give significant attention to WHR. From the perspective of Epidemiology and public health, this study highlights the importance of quitting smoking and maintaining healthy weight and provides a reference for the prevention and treatment of colorectal cancer in the future.

## Conclusion

Our MR analysis indicates a causal association between a higher waist-to-hip ratio and the risk of colorectal cancer and a suggestive association between smoking and the risk of colorectal cancer among Europeans.

### Supplementary Information

Supplementary Information 1.

Supplementary Information 2.

Supplementary Information 3.

Supplementary Information 4.

Supplementary Information 5.

Supplementary Information 6.

Supplementary Information 7.

Supplementary Information 8.

Supplementary Information 9.

Supplementary Information 10.

Supplementary Information 11.

Supplementary Figures.

## Data Availability

The exposure datasets analyzed during this study are included in its supplementary information files. The datasets of outcome analyzed during the current study are available in the [FinnGen] repository, [https://www.finngen.fi/].

## Abbreviations

MR: Mendelian randomization
RCTs: Randomized controlled trials
CRC: Colorectal cancer
SNP: Single nucleotide polymorphisms
IVs: Instrumental variables
GWAS: Genome-wide association study
BMI: Body mass index
WHR: Waist-to-hip ratio
IVW: Inverse variance weighted
IARC: International agency for research on cancer

## References

[1] Bray F (2018). Global cancer statistics 2018: GLOBOCAN estimates of incidence and mortality worldwide for 36 cancers in 185 countries. CA Cancer J. Clin..

[2] Sung H (2021). Global Cancer Statistics 2020: GLOBOCAN Estimates of Incidence and Mortality Worldwide for 36 Cancers in 185 Countries. CA Cancer J. Clin..

[3] Dekker, E., Tanis, P. J., Vleugels, J. L. A., Kasi, P. M. & Wallace, M. B. Colorectal cancer. **394**, 1467–1480 (2019). 10.1016/S0140-6736(19)32319-0 31631858

[4] Davidson KW (2021). Screening for colorectal cancer. JAMA.

[5] Kucuk O (2022). Walk more, eat less, don’t stress. Cancer Epidemiol. Biomark. Prev..

[6] Jo A, Oh H (2019). Incidence of colon cancer related to cigarette smoking and alcohol consumption in adults with metabolic syndrome: prospective cohort study. J. Kor. Acad. Nurs..

[7] Kontou N (2012). Alcohol consumption and colorectal cancer in a mediterranean population. Dis. Colon Rectum.

[8] Smith GD, Hemani G (2014). Mendelian randomization: genetic anchors for causal inference in epidemiological studies. Hum. Mol. Genet..

[9] Gala H, Tomlinson I (2020). The use of Mendelian randomization to identify causal cancer risk factors: Promise and limitations. J. Pathol..

[10] Bochud M, Rousson V (2010). Usefulness of Mendelian randomization in observational epidemiology. Int. J. Environ. Res..

[11] Pulit SL (2018). Meta-analysis of genome-wide association studies for body fat distribution in 694 649 individuals of European ancestry. Hum. Mol. Genet..

[12] Liu M (2019). Association studies of up to 1.2 million individuals yield new insights into the genetic etiology of tobacco and alcohol use. Nat. Genet..

[13] Klimentidis YC (2018). Genome-wide association study of habitual physical activity in over 377,000 UK Biobank participants identifies multiple variants including CADM2 and APOE. Int. J. Obes..

[14] Pierce BL, Ahsan H, VanderWeele TJ (2010). Power and instrument strength requirements for Mendelian randomization studies using multiple genetic variants. Int. J. Epidemiol..

[15] Boef AGC, Dekkers OM, Cessie S (2015). Mendelian randomization studies: A review of the approaches used and the quality of reporting. Int. J. Epidemiol..

[16] Hemani G, Bowden J, Smith GD (2018). Evaluating the potential role of pleiotropy in Mendelian randomization studies. Hum. Mol. Genet..

[17] Bowden J, Smith GD, Haycock PC, Burgess S (2016). Consistent estimation in mendelian randomization with some invalid instruments using a weighted median estimator. Genet. Epidemiol..

[18] Xue H, Shen X, Pan W (2021). Constrained maximum likelihood-based Mendelian randomization robust to both correlated and uncorrelated pleiotropic effects. Am. J. Hum. Genet..

[19] Mizoue T (2006). Tobacco smoking and colorectal cancer risk: An evaluation based on a systematic review of epidemiologic evidence among the japanese population. Jpn. J. Clin. Oncol..

[20] Thomson B (2020). Association of childhood smoking and adult mortality: Prospective study of 120,000 Cuban adults. Global.

[21] Botteri E (2008). Smoking and colorectal cancer. JAMA.

[22] Bai X (2022). Cigarette smoke promotes colorectal cancer through modulation of gut microbiota and related metabolites. Gut.

[23] McNabb S (2019). Meta-analysis of 16 studies of the association of alcohol with colorectal cancer. Int. J..

[24] Tabung FK (2018). Association of dietary inflammatory potential with colorectal cancer risk in men and women. JAMA Oncol..

[25] Wang Q, Zhou W (2021). Roles and molecular mechanisms of physical exercise in cancer prevention and treatment. J. Sport Sci..

[26] Murray JM, Coleman HG, Hunter RF (2020). Physical activity and cancer risk: Findings from the UK Biobank, a large prospective cohort study. Epidemiology.

[27] Kim SY, Yoo DM, Min C, Choi HG (2021). Association between coffee consumption/physical exercise and gastric, hepatic, colon, breast, uterine cervix, lung, thyroid, prostate, and bladder cancer. Nutrients.

[28] Lee HH (2023). Association between regular physical activity and lower incidence of colorectal cancer in patients with diabetes mellitus: A nationwide cohort study. Disease.

[29] Ahmadi MN (2022). Vigorous physical activity, incident heart disease, and cancer: how little is enough?. Eur. J..

[30] Stamatakis E (2023). Vigorous intermittent lifestyle physical activity and cancer incidence among nonexercising adults. JAMA Oncol..

[31] Center MM, Jemal A, Smith RA, Ward E (2009). Worldwide Variations in Colorectal Cancer. CA Cancer J. Clin..

[32] Arnold M (2020). Global burden of 5 major types of gastrointestinal cancer. Gastroenterology.

[33] Khan I (2023). Surrogate adiposity markers and mortality. JAMA Netw. Open.

[34] Lega IC, Lipscombe LL (2019). Review: Diabetes, obesity, and cancer—pathophysiology and clinical implications. Endocr. Rev..

[35] Tang B (2017). Waist-hip Ratio (WHR), a better predictor for prostate cancer than body mass index (BMI): Results from a Chinese hospital-based biopsy cohort. Sci. Rep..

[36] Bao X (2022). Proteomic profiles of body mass index and waist-to-hip ratio and their role in incidence of diabetes. J. Clin. Endocrinol. Metab..

[37] Schult A, Mehlig K, Björkelund C, Wallerstedt S, Kaczynski J (2017). Waist-to-hip ratio but not body mass index predicts liver cirrhosis in women. Scand. J. Gastroenterol..

[38] Lee HJ (2015). Waist-to-hip ratio is better at predicting subclinical atherosclerosis than body mass index and waist circumference in postmenopausal women. Maturitas.

[39] Ringhofer C (2017). Waist to hip ratio is a better predictor of esophageal acid exposure than body mass index. Neurogastroenterol. Motil..

[40] Ke J-F (2022). Waist-to-height ratio has a stronger association with cardiovascular risks than waist circumference, waist-hip ratio and body mass index in type 2 diabetes. Res. Clin. Pract..

[41] Li H (2012). Body weight, fat distribution and colorectal cancer risk: A report from cohort studies of 134 255 Chinese men and women. Int. J. Obes..

