# Supplementary material for: Unhealthy lifestyle factors and the risk of colorectal cancer: a Mendelian randomization study
Source: Sci Rep. 2024 Jun 15;14:13825. doi: 10.1038/s41598-024-64813-y (PMC11180165; doi:10.1038/s41598-024-64813-y)
Supplement: Supplementary file 12 — Supplementary Figures. [file 41598_2024_64813_MOESM12_ESM.docx]

**Figure S1.** The results of “leave-one-out method” sensitivity analysis of the causal effect of lifestyle factors on colorectal cancer risk


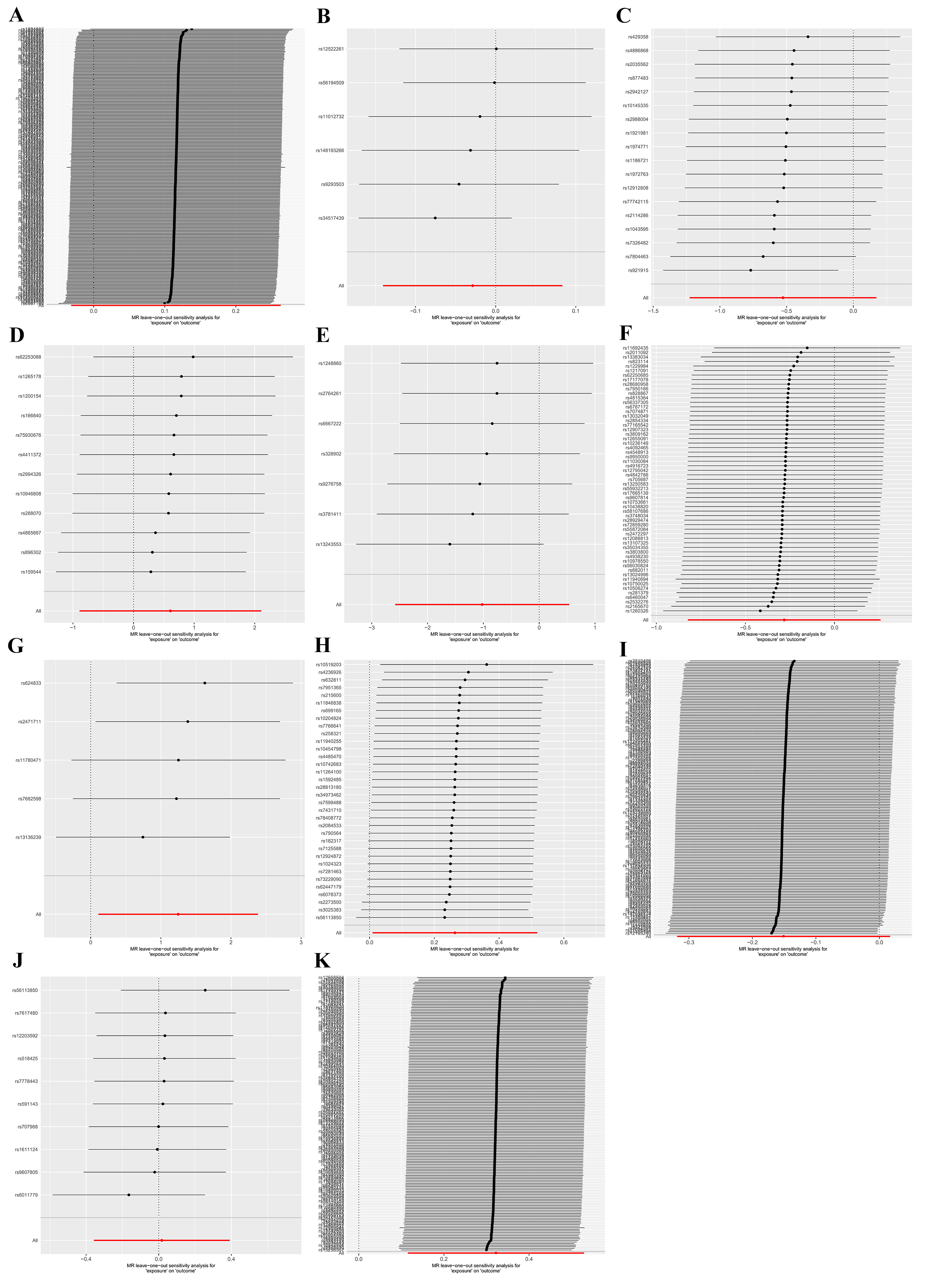


A: BMI: Body mass index; B: AccAve: Accelerometer-based physical activity measurement (average acceleration); C: MVPA: moderate-to-vigorous physical activity; D: SSOE: strenuous sports or other exercise; E: VPA: vigorous physical activity; F: DrnkWk: Available measures of alcohol use were simpler, with drinks per week; G: AgeSmk: age of initiation of regular smoking; H: CigDay: Heaviness of smoking was measured with cigarettes per day; I: SmkInit: a binary phenotype indicating whether an individual had ever smoked regularly; J: SmkCes: Smoking cessation; K: WHR: Waist-to-hip ratio

**Figure S2.** The forest plots depict causal association between other exposures and colorectal cancer


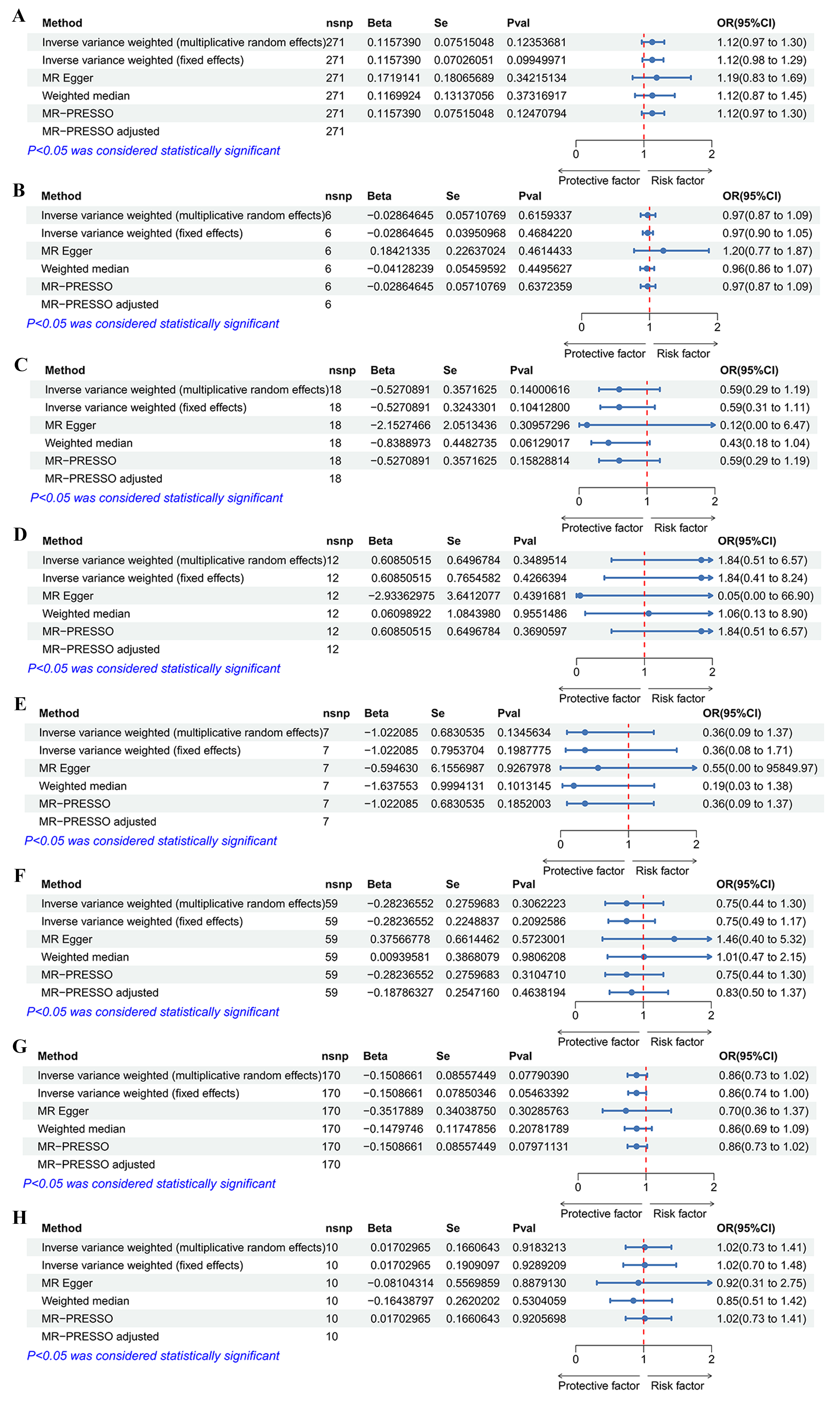


A: BMI: Body mass index; B: AccAve: Accelerometer-based physical activity measurement (average acceleration); C: MVPA: moderate-to-vigorous physical activity; D: SSOE: strenuous sports or other exercise; E: VPA: vigorous physical activity; F: DrnkWk: Available measures of alcohol use were simpler, with drinks per week; G: SmkInit: a binary phenotype indicating whether an individual had ever smoked regularly; H: SmkCes: Smoking cessation

**Figure S3.** The funnel plots for evaluating heterogeneity


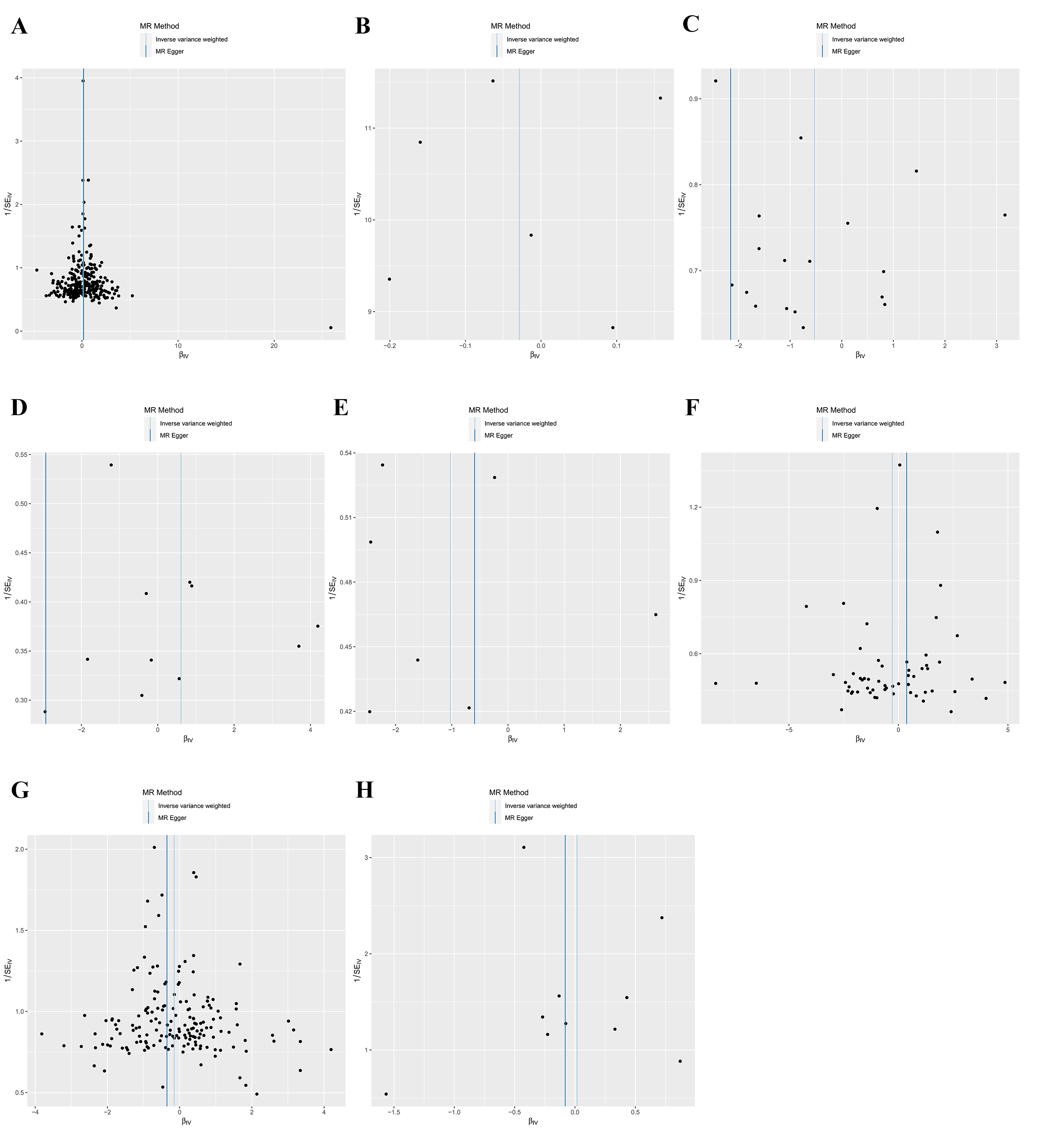


A: BMI: Body mass index; B: AccAve: Accelerometer-based physical activity measurement (average acceleration); C: MVPA: moderate-to-vigorous physical activity; D: SSOE: strenuous sports or other exercise; E: VPA: vigorous physical activity; F: DrnkWk: Available measures of alcohol use were simpler, with drinks per week; G: SmkInit: a binary phenotype indicating whether an individual had ever smoked regularly; H: SmkCes: Smoking cessation

**Figure S4.** The Scatter plots depict causal association between other exposures and colorectal cancer


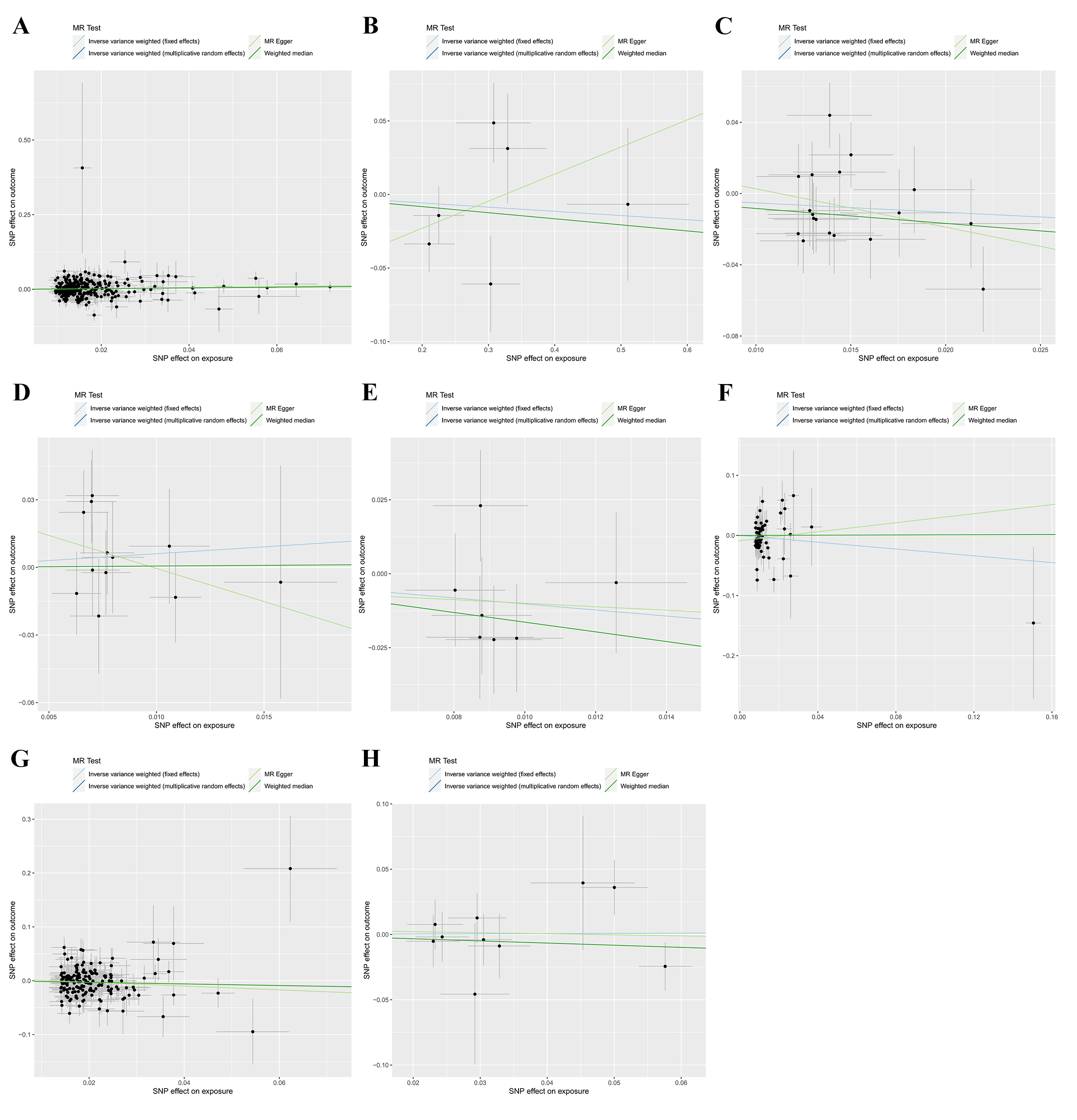


A: BMI: Body mass index; B: AccAve: Accelerometer-based physical activity measurement (average acceleration); C: MVPA: moderate-to-vigorous physical activity; D: SSOE: strenuous sports or other exercise; E: VPA: vigorous physical activity; F: DrnkWk: Available measures of alcohol use were simpler, with drinks per week; G: SmkInit: a binary phenotype indicating whether an individual had ever smoked regularly; H: SmkCes: Smoking cessation
